# Exploring the fate of *Listeria monocytogenes* in an *in vitro* digestion and fecal fermentation model: insights into survival during digestion and interaction with gut microbiota

**DOI:** 10.3389/fmicb.2025.1616720

**Published:** 2025-07-23

**Authors:** Dong Woo Kim, Saloni Singh, Ui In Kim, So Hyeon An, Hyeon Ji Je, Dong Young Lee, Eun Ju Yun, Ok Kyung Koo

**Affiliations:** ^1^Department of Food Science & Technology, Chungnam National University, Daejeon, Republic of Korea; ^2^Department of Biotechnology, The Catholic University of Korea, Bucheon, Republic of Korea

**Keywords:** *Listeria monocytogenes*, gut microbiota, *in vitro* digestion, fecal fermentation, microbial interactions

## Abstract

*Listeria monocytogenes* is a foodborne pathogen that causes listeriosis, a disease with a mortality rate of 20 ~ 30%. This bacterium enters the human body through contaminated food or ingredients and encounters primary innate defense systems, including gastric acid, bile salts, and gut microbiota. These systems play a critical role in preventing pathogen colonization and infection. However, interactions with pathogens can also alter the gut microbiota profile. This study aimed to investigate the host’s defense mechanisms against *L. monocytogenes* and the changes in the gut microbiota profile following infection. *L. monocytogenes* ATCC 7644 showed the greatest reduction (7.6 log CFU), followed by ATCC 19111 (5.71 log), F2365 (5.02 log), ATCC 19113 (3.96 log), and NCCP 14714 (3.38 log), while the pooled cocktail exhibited a 3.49 log CFU reduction. Notably, the clinical isolates NCCP 14714 and F2365 exhibited greater resistance to the simulated digestive process compared to the food isolate ATCC 7644. *L. monocytogenes* infection induced notable shifts in specific bacterial groups, including *Bacteroides*, *Bifidobacterium*, and the *Mediterraneibacter gnavus* group, as well as an increase in ethanol levels. These alterations may contribute to gut barrier disruption and the upregulation of immune responses, ultimately promoting the pathogenesis of *L. monocytogenes* infection. The findings from this study provide valuable insights into the interaction between *L. monocytogenes* and the human gut microbiota, offering a comparative reference for the interpretation of future research.

## 1 Introduction

*Listeria monocytogenes* is a Gram-positive, foodborne pathogen renowned for its capacity to cause severe infections, termed listeriosis (Osek et al., 2022). Listeriosis is recognized as one of the top five foodborne illnesses, with a mortality rate of 20 ~ 30% (Koopmans et al., 2023; Osek et al., 2022). The illness primarily occurs in pregnant women, newborns, elderly, and immunocompromised individuals, with pregnant women being over 100 times more likely to develop the infection compared to women with reproductive capability (Centers for Disease Control and Prevention, 2024; Koopmans et al., 2023). The incidence of foodborne outbreaks caused by *L. monocytogenes* has decreased through the implementation of regulations, practices, and screening methods such as HACCP (Koopmans et al., 2023). However, the costs associated with listeriosis still reached up to 22 billion dollars in North America, serving as a significant public health concern worldwide (Koopmans et al., 2023).

*Listeria monocytogenes* can primarily be transmitted through contaminated food ingredients or food products, such as meat, fish, ready-to-eat products, sliced vegetables, juice, and salad (Buchanan et al., 2017; Je et al., 2024). *L. monocytogenes* is introduced into the digestive system along with these contaminated foods, where it encounters primary defense mechanisms in the human body: gastric acid and bile salts (Koopmans et al., 2023). These harsh conditions of the digestive system can structurally damage the bacterial surface, increasing surface irregularities and potentially causing dissolution during passage through the gastrointestinal tract (Zou et al., 2024). Bile hinders microbial growth, and its toxicity against bacteria leads to heightened DNA damage, formation of secondary RNA structures, and instability in cellular membranes (Dowd et al., 2011). However, some *L. monocytogenes* can overcome acidic and enzymatic stress through the glutamate decarboxylase system, acid tolerance response, bile acid deconjugation, and the upregulation of multidrug efflux pumps (Koopmans et al., 2023; Zou et al., 2024). The σ^B^ operon is also considered a critical genetic element in adapting to acidic stress and surviving in the gastrointestinal tract (Gahan and Hill, 2014; Guerreiro et al., 2022). *L. monocytogenes*, which survives the digestive process, reaches the intestine, where it adheres to or invades the intestinal epithelium, leading to infection in the host (Koopmans et al., 2023).

Gut microbiota, comprising Bacteroidota (formerly Bacteroidetes), Bacillota (formerly Firmicutes), Pseudomonadota (formerly Proteobacteria), and Actinomycetota (formerly Actinobacteria), colonize the human intestine and play a crucial role in maintaining homeostasis (Park and Im, 2020). They are resistant against pathogens by competing with limited resources, altering the gut environment, and secreting antimicrobial substances such as short-chain fatty acids (SCFAs) and bacteriocins (Kamada et al., 2013). Some *Bacteroides* and lactic acid bacteria exhibit direct colonization resistance and infection resistance against pathogens such as *Citrobacter rodentium*, *Escherichia coli*, *Clostridium difficile,* and *Salmonella* (Buffie and Pamer, 2013). SCFAs decrease pH-sensitive pathogens, such as *Enterobacteriaceae* and Clostridia, and reduce the pathogenicity of *Campylobacter jejuni* and *Staphylococcus aureus* (Alva-Murillo et al., 2012; Sittipo et al., 2019; Van Deun et al., 2008). Pathogens can also lead to the death of anaerobic microbes and modify the gut microbiota profile by inducing secretion of antibacterial compounds, such as reactive oxygen species and reactive nitrogen species (Bäumler and Sperandio, 2016; Rogers et al., 2021). These imbalances reduce the diversity of gut microbiota and metabolite production, which aid pathogens in evading the microbial defense mechanisms (Rogers et al., 2021).

Various studies have explored pathogenic susceptibility, survival rates, and dynamics of intestinal bacteria in animal models (Becattini et al., 2017; Las Heras et al., 2019; Wolter et al., 2021). However, significant biological differences in intestinal structures, immune systems, and particularly in gut microbiota profiles between animals and humans can lead to results that may not accurately reflect human conditions (Park and Im, 2020). An *in vitro* fecal fermentation model, which utilizes human fecal inoculation and maintains a stable gut microbiota composition over extended periods, offers a more relevant platform for studying bacteria-to-bacteria interactions between human gut microbiota and *L. monocytogenes* (Li et al., 2019). Additionally, *in vitro* studies offer several advantages, including speed, cost-effectiveness, reduced labor demands, the ability to process multiple samples simultaneously, and no ethical concerns (Li et al., 2019; Minekus et al., 2014).

The aim of this study was to investigate the host’s defense strategy against *L. monocytogenes* and the modulation of the gut microbiota profile following listerial infection. The survival of *L. monocytogenes* during digestion was assessed *in vitro*, and the changes in gut microbiota and their metabolites induced by the pathogen were explored using a fecal fermentation model (Li et al., 2019; Minekus et al., 2014). Ultimately, understanding the interactions between *L. monocytogenes* and the gut microbiota is crucial for developing effective strategies to enhance the host’s defense mechanisms. Insights from this study may enhance our understanding of the interactions between gut microbiota and *L. monocytogenes*, aiming to mitigate the impact of *L. monocytogenes* infection and thereby promoting overall gut health and food safety.

## 2 Materials and methods

### 2.1 Selection of *L. monocytogenes* strains and growth conditions

Five *L. monocytogenes* strains [*L. monocytogenes* F2365 (4b), ATCC 19111 (1/2a), ATCC 19113 (3a), ATCC 7644 (1/2c), and NCCP 14714 (1/2b)] were selected from the culture collection at the Food Safety Lab of Chungnam National University, South Korea. These strains were chosen to represent a diverse range of serotypes commonly associated with foodborne outbreaks and environmental isolates (Koopmans et al., 2023). All strains were stored at −80°C. For each experiment, the strains were incubated on brain heart infusion (BHI; Kisan Bio, Seoul, South Korea) agar at 37°C for 24 h. Subsequently, a single colony of each *L. monocytogenes* was inoculated into BHI broth and cultured overnight at 37°C. A pooled *L. monocytogenes* culture was prepared by combining equal proportions of each strain incubated in broth.

### 2.2 *In vitro* digestion model

The survival of *L. monocytogenes* during gastrointestinal transit was assessed using an *in vitro* digestion model, with modifications based on the protocol of Pettersen et al. (2019). The five *L. monocytogenes* strains described above were tested with pooled cocktail to represent the genetic and phenotypic diversity in nature and compared the result with each single-strain. Overnight cultures of *L. monocytogenes* strains were centrifuged, and the pellets were resuspended in 0.1% peptone water (Kisan Bio). The bacterial suspension was exposed to simulated gastric fluid (SGF) containing porcine pepsin (2,000 U/mL; Sigma-Aldrich, St. Louis, MO, United States) in a 1:1 ratio. The SGF composition included 6.9 mM KCl (Junsei, Tokyo, Japan), 0.9 mM KH_2_PO_4_ (Sigma-Aldrich), 25 mM NaHCO_3_ (Daejung, Siheung, South Korea), 47.2 mM NaCl (Daejung), 0.1 mM MgCl_2_·7H_2_O (Daejung), 0.5 mM (NH_4_)_2_·CO_3_ (Junsei), and 0.15 mM CaCl_2_·2H_2_O (Daejung). The pH was adjusted to either 2.0 or 5.5 using 1 M HCl (Daejung) to mimic fasted or fed states (AquaSearcher AB23PH; Ohaus Corporation, Parsippany, NJ, United States). The gastric mixtures were incubated at 37°C in a shaking water bath (Maxturdy-18, Daihan Scientific, South Korea) at 100 rpm for 40 min. For the intestinal phase, the gastric mixture was combined with a simulated intestinal fluid (SIF), supplemented with 10 mM bovine bile (Sigma-Aldrich), and 100 U/mL porcine pancreatin (Sigma-Aldrich) at a 1:1 ratio. The SIF composition included 6.8 mM KCl, 0.8 mM KH_2_PO_4_, 85 mM NaHCO_3_, 38.4 mM NaCl, 0.33 mM MgCl_2_·6H_2_O, and 0.6 mM CaCl_2_·2H_2_O. The pH was adjusted to 7.0 using 1 M NaOH (Daejung) and incubated at 37°C in a shaking water bath at 100 rpm for 2 h. At each step, *L. monocytogenes* was enumerated by serial dilution in 0.1% peptone water and plating onto Oxford agar (Kisan Bio).

### 2.3 Fecal sample collection

Fecal samples (~5 g) were collected from three healthy donors using stool containers (SPL Life Sciences, Pocheon, South Korea). The samples were stored at 4°C and processed within 6 h. The donors were selected based on the absence of congenital or chronic diseases, no medication, and no antibiotic treatment within 4 weeks prior to collection. The ethical approval for fecal sample collection was obtained from the Institutional Review Board (IRB, 202209-BR-125-01).

### 2.4 *In vitro* fecal fermentation model

An *in vitro* fecal fermentation was conducted, based on Li et al. (2019) with slight modifications. Fresh fecal samples (2 g) were homogenized using a vortexer in 20 mL of sterile phosphate-buffered saline (PBS; Gibco BRL, Paisley, Scotland, UK) with 0.5% (w/v) L-cysteine hydrochloride (Junsei). The homogenized fecal mixture was filtered through sterile gauze. All experiments were conducted in a Coy Anaerobic Vinyl chamber (Coy Laboratory Product, MI, United States) under an anaerobic atmosphere (5% H_2_, 5% CO_2_, and 90% N_2_) at 37°C.

MiPro medium was prepared with the following components: 2.0 g/L peptone water, 2.0 g/L yeast extract (Kisan Bio), 0.5 g/L L-cysteine hydrochloride, 2 mL/L Tween 80 (Daejung), 5 mg/L hemin bovine (Sigma-Aldrich), 10 μL/L vitamin K1 (Sigma-Aldrich), 0.4 g/L K_2_HPO_4_ (Sigma-Aldrich), 0.4 g/L KH_2_PO_4_, 0.1 g/L MgSO_4_·7H_2_O, 0.1 g/L CaCl_2_·2H_2_O, 4.0 g/L NaHCO_3_, 4.0 g/L porcine gastric mucin (Sigma-Aldrich), and 0.5 g/L bile salts (Merck, Darmstadt, Germany) (Li et al., 2019). The sterile medium was placed in the anaerobic chamber a day prior to the experiment to ensure anaerobic conditions. Overnight cultures of *L. monocytogenes* were centrifuged and resuspended in fresh MiPro medium. The *L. monocytogenes* cocktail and the gut microbiota inocula were adjusted to reach approximately 8 log gene copies (GC)/mL. Fermentation was conducted in 96-deep well plates (Eppendorf, Hamburg, Germany) covered with silicone mats punctured for sampling, and incubated at 37°C. The samples were collected at 6 h, 12 h, and 24 h, and pH was measured at each time point. The survival of *L. monocytogenes* was verified by plating onto Oxford agar. The pellets were collected via centrifugation at 8,000 rpm for 10 min and stored at −80°C for subsequent analysis. The supernatants were filtered using a 0.22 μm syringe filter (13 mm; polytetrafluoroethylene) (Advantec, Tokyo, Japan) and stored at −80°C for SCFA analysis.

### 2.5 Quantitative PCR, and 16S rRNA amplicon sequencing

DNA was extracted using a NucleoSpin DNA Stool kit (Macherey-Nagel, Duren, Germany). Quantitative PCR (qPCR) was conducted on a CFX Connect Real-Time System (Bio-Rad, Hercules, CA, United States), using iQ SYBR-Green supermix (Bio-Rad) with 0.5 μM forward and reverse primers and template DNA. Universal primers targeting the 16S rRNA gene (forward: 5`-GTG STG CAY GGY YGT CGT CA-3`; reverse: 5`-ACG TCR TCC MCN CCT TCC TC-3`) were used following cycling conditions: 95°C for 3 min followed by 40 cycles of 95°C for 5 s, and 60°C for 30 s (Fuller et al., 2007; Reichardt et al., 2018). The 16S rRNA gene of *Bifidobacterium miconisargentati* 82T25 (accession ID: NR_181805.1, 148 bp) was used as the standard with concentrations ranging from 10^4^ to 10^9^ GC/ng (Macrogen Co. Ltd., Seoul, South Korea).

The 16S rRNA amplicon sequencing of the gut microbiota was conducted by Sanigen Co. Ltd. (Anyang, South Korea). In short, PCR was conducted with 341F and 806R primers to amplify the V3-V4 region of bacterial 16S rRNA genes. Then, the amplicon purification was carried out employing AMPure XP beads (Beckman Coulter, CA, United States). Library preparation for sequencing was carried out using the Nextera XT library prep kit (Illumina, San Diego, CA, United States). Sequencing was performed on the Illumina MiSeq platform (Illumina), generating 2 × 300 bp paired-end reads. Sequence processing, including trimming of low-quality reads, error correction of noisy reads, and elimination of chimeric sequences, was performed with Trimmomatic v0.39 (Bolger et al., 2014) and DADA2-QIIME2 (Callahan et al., 2016). Taxonomic classification was conducted using the SILVA 138 reference database (Quast et al., 2012), and diversity analyses were performed with QIIME2 (Bolyen et al., 2019). Metadata from this study is publicly available through the NCBI Sequence Read Archive under BioProject accession number PRJNA1254141, with associated BioSample accessions SAMN48096620 to SAMN48096647. To clearly understand the effects of *L. monocytogenes* infection, the OTU value of *L. monocytogenes* was excluded prior to analyzing diversity and profile abundance.

### 2.6 Quantitative analysis of SCFAs

Short-chain fatty acids concentrations in the fermentation supernatants were determined using the Nexera series high-performance liquid chromatography (HPLC) system (Shimadzu, Kyoto, Japan) installed with a RID-20A detector (Shimadzu) and an Aminex HPX-87H column (300 × 7.8 mm; Bio-Rad). The mobile phase consisted of 0.008 N sulfuric acid (Sigma-Aldrich), maintained at a constant flow rate of 0.6 mL/min at 35°C. Sample 20 μL was injected, and the analytes were detected over a 25 min run time. All samples were treated under the same conditions.

### 2.7 Statistical analysis

All experiments were conducted in triplicate to ensure data reproducibility and reliability. Statistical analysis was performed using one-way analysis of variance (ANOVA), followed by Tukey’s multiple comparisons test. A *p-*value of <0.05 was considered statistically significant for all analyses.

## 3 Results

### 3.1 Survival of *L. monocytogenes* during digestion

**Figure 1:**
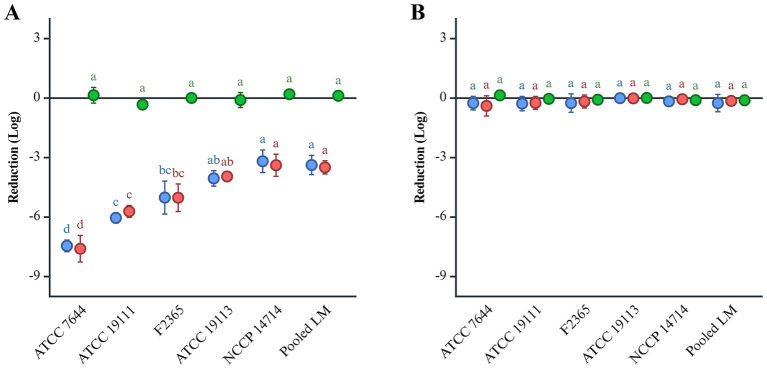
The reduction of *L. monocytogenes* at pH 2.0 **(A)** and 5.5 **(B)** during *in vitro* gastric digestion. *L. monocytogenes* was inoculated at a final concentration of 9 log CFU/mL. Gastric and intestinal digestions are indicated in red and green, respectively. The overall reduction is shown in blue. Significant differences (*p* < 0.05) are indicated by alphabets.

Five individual *L. monocytogenes* strains and their cocktail were subjected to an *in vitro* digestion model. During the gastric phase at pH 2.0, the five individual strains exhibited an average reduction of 5.13 log CFU, while the pooled *L. monocytogenes* cocktail demonstrated a 3.49 log CFU reduction (Figure 1A). Log reduction rates varied across individual strains, ranging from 3.38 to 7.6. *L. monocytogenes* ATCC 7644 showed the highest reduction at 7.6 log, followed by ATCC 19111 with 5.71 log, F2365 with 5.02 log, ATCC 19113 with 3.96 log, and ATCC 14714 with 3.38 log reduction. In the gastric phase at pH 5.5, all strains showed no significant differences in survival (Figure 1B). In the subsequent intestinal phase, regardless of initial pH, survival of all strains remained consistent within a ± 1 log range, indicating stable resistance throughout this phase (Figure 1).

### 3.2 Survival of gut microbiota and *L. monocytogenes* during fecal fermentation

Gut microbiota in the *L. monocytogenes*-infected and non-infected samples reached 10.62 and 10.25 log GC/mL at 6 h, respectively (Supplementary Figure S1). The levels further increased to 11.39 and 11.46 log GC/mL at 12 h, then stabilized at 11.61 and 11.65 log GC/mL at 24 h. The *L. monocytogenes* cocktail, 7.34 log CFU/mL, increased to 8.58 log CFU/mL at 6 h, stabilized at 8.55 log CFU/mL at 12 h, and slightly declined to 8.17 log CFU/mL by 24 h. When cultured in MiPro medium alone, *L. monocytogenes* reached 8.41 log CFU/mL at 6 h, rose to 8.61 log CFU/mL at 12 h, and decreased slightly to 8.47 log CFU/mL by 24 h. Initial pH was 7.6, which decreased to 6.98 at 6 h, remained steady until 12 h, and then slightly increased to 7.2 by 24 h.

### 3.3 Dynamics of gut microbiota profile

**Figure 2:**
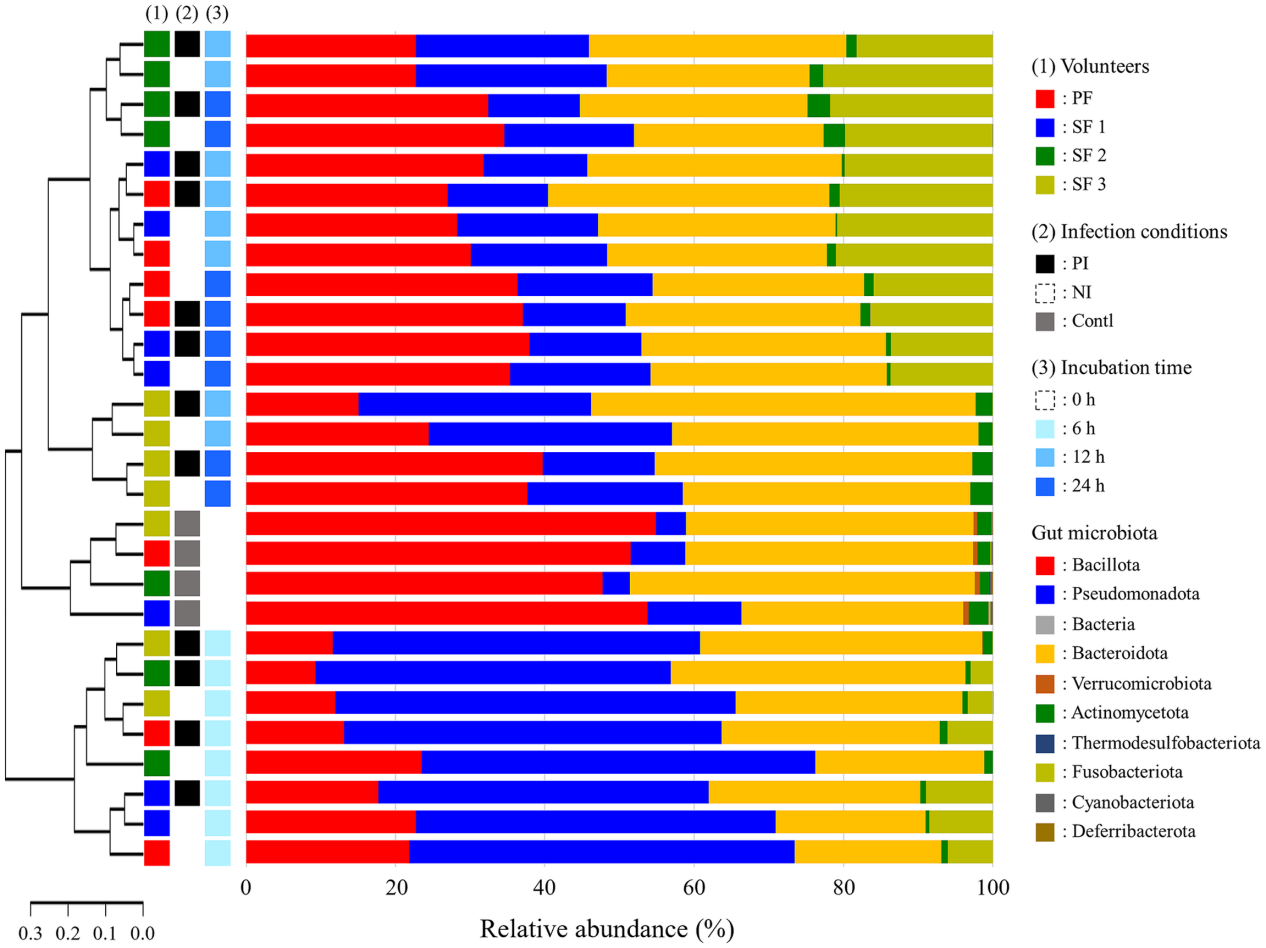
Phylogenetic tree and relative abundance of gut microbiota at the phylum level after *L. monocytogenes* infection during fecal fermentation, categorized by (1) volunteers, (2) *in vitro* infection conditions, and (3) incubation time. The phylogenetic analysis was performed using weighted distance values of gut microbiota. PF, pooled feces; SF, single feces; PI, post-infected; NI, non-infected; Contl, control.

At 0 h, the dominant phyla in fecal samples were Bacillota (51.98%), Bacteroidota (38.21%), Pseudomonadota (6.89%), and Fusobacteriota (0.11%) comprising over 95% of the microbial community (Figure 2). At 6 h, there was a significant shift in the microbial composition: Pseudomonadota and Fusobacteriota increased to 49.72 and 4.47%, respectively, while Bacteroidota and Bacillota decreased to 28.43 and 16.46%, respectively. Pseudomonadota reduced by 22.12% at 12 h and by 16.40% at 24 h, while Bacteroidota and Bacillota recovered to 35.90 and 25.26% at 12 h, respectively, with further stabilization at 24 h. Fusobacteriota increased to 15.39% at 12 h and remained relatively stable at 12.66% at 24 h. Notably, the abundance of Fusobacteriota in single feces 3 (SF3) decreased to below 0.05% after 12 h.

**Figure 3:**
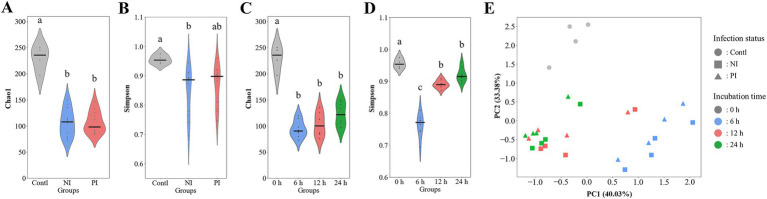
Violin plots and principal coordinate analysis (PCA) comparing the alpha and beta diversity of gut microbiota during fecal fermentation based on infection status **(A,B)** and incubation time **(C,D)**. The plots include the species richness as measured by the chao1 index **(A,C)** as well as the species evenness as measured by the Simpson diversity index **(B,D)**. PCA is presented by weighted Bray–Curtis distances **(E)**. Significant differences (*p* < 0.05) are indicated by alphabets. Contl, control; NI, non-infected; PI, post-infected.

Alpha and beta diversity analyses indicated that infection with *L. monocytogenes* reduced richness while increased evenness, though the changes were not statistically significant (Figures 3A,B). The diversity was significantly decreased at 6 h, recovered at 12 h, with richness and evenness continuing to increase through 24 h (Figures 3C,D). Weighted distance analysis displayed clustering differences based on infection conditions, with incubation time exerting a stronger influence on cluster patterns (Figure 3E). Notably, 0 h samples were distinct from latter time points, while 12 h and 24 h samples exhibited greater proximity, clustering more closely together, which aligns with phylogenetic results (Figure 2).

### 3.4 Gut microbiota profile in the phylum, family, and genus level

**Figure 4:**
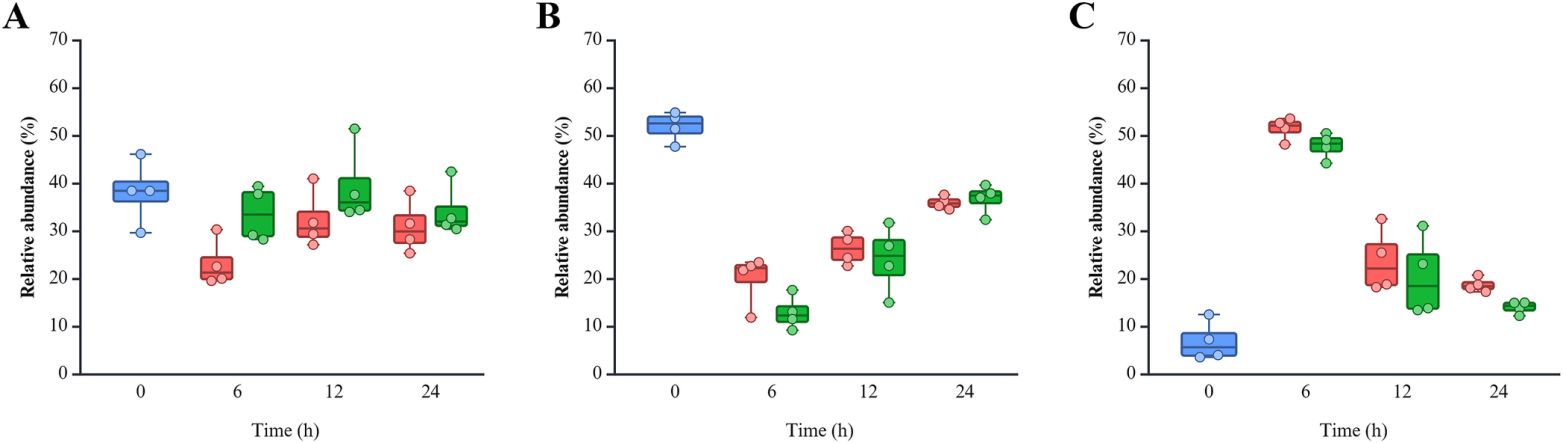
Relative abundance of three phyla of interest: **(A)** Bacteroidota, **(B)** Bacillota, and **(C)** Pseudomonadota. The color of each bar chart represents different infection conditions: control (blue), non-infected (red), and post-infected (green). Each dot represents the value for each sample.

**Figure 5:**
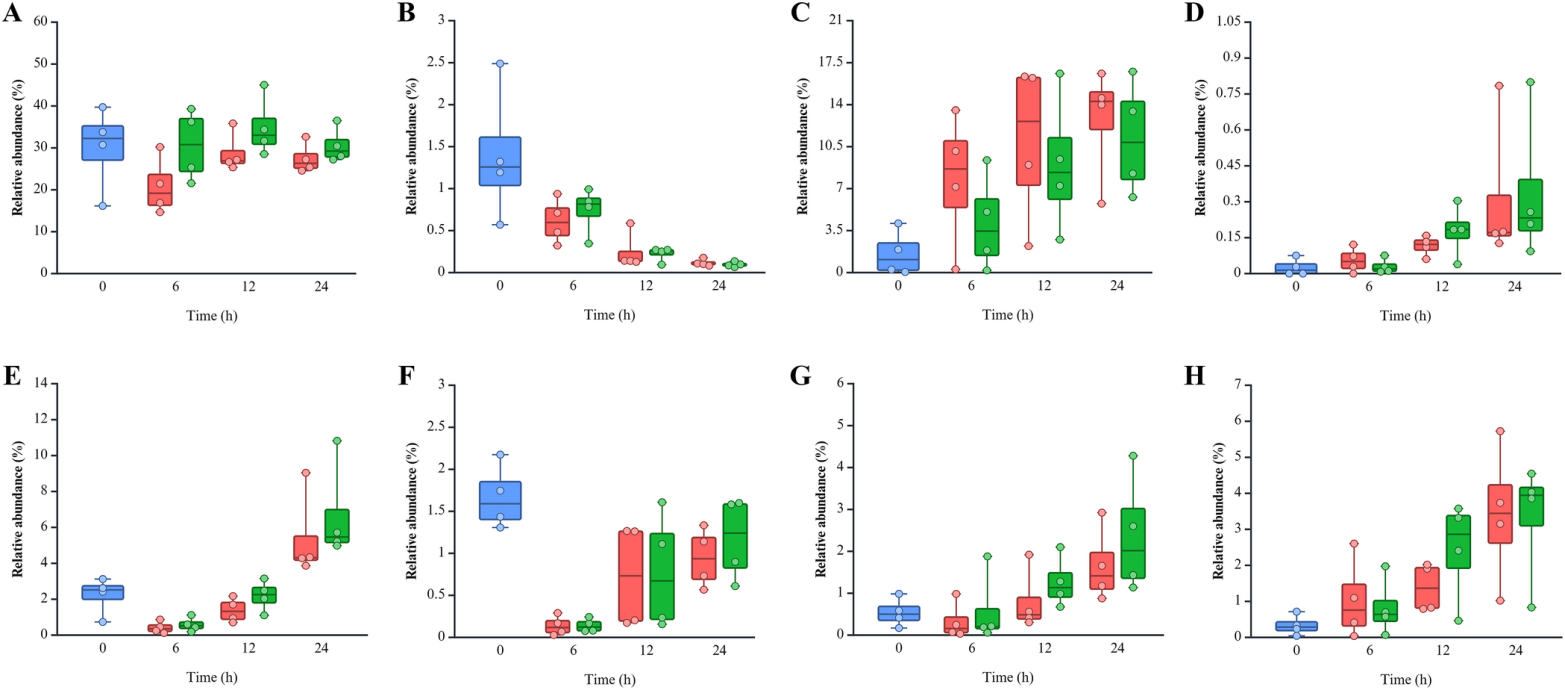
Relative abundance of eight genera belonging to *Bacteroidaceae*, *Bifidobacteriaceae*, *Erysipelotrichaceae*, and *Lachnospiraceae*: **(A)** *Bacteroides*, **(B)** *Bifidobacterium*, **(C)** *Faecalitalea*, **(D)** *Clostridium innocuum* group, **(E)** *Lachnoclostridium*, **(F)** *Blautia*, **(G)** *Ruminococcus torques* group, and **(H)** *Mediterraneibacter gnavus* group. The color of each bar chart represents different infection conditions: control (blue), non-infected (red), and post-infected (green). Each dot represents the value for each sample.

Pseudomonadota was more dominant in the non-infected (NI) group (18.77 ~ 51.54%) than post-infected (PI) groups (14.03 ~ 47.90%), while Bacteroidota was more abundant in PI (33.69 ~ 39.43%) compared to NI (23.17 ~ 32.37%) groups (Figure 4). This pattern remained consistent across all fecal samples and time points. Bacillota showed higher levels in NI (20.00%) than PI (12.93%) at 6 h, with similar levels thereafter. *L. monocytogenes* infection delayed the recovery of Bacillota at 6 h in the pooled feces (PF) group and SF1 and 2 groups and 12 h in the SF3 group (data not shown). At the family level, PI groups consistently exhibited lower *Erysipelotrichaceae* but higher *Bacteroidaceae*, *Lachnospiraceae*, and *Bifidobacteriaceae* abundances than NI at all time points (Supplementary Figure S2). Notably, both *Bacteroidaceae* and *Lachnospiraceae* showed a marked increase in response to *L. monocytogenes* infection across all fecal groups (data not shown). Eight genera within these families exhibited abundance patterns consistent with trends observed at the family level (Figure 5). *Bacteroides* (*Bacteroidaceae*) and *Bifidobacterium* (*Bifidobacteriaceae*) were more abundant in PI (30.55 ~ 34.90% and 0.10 ~ 0.74%) than NI (20.80 ~ 28.74% and 0.12 ~ 0.61%). *Faecalitalea*, belonging to *Erysipelotrichaceae*, was more prevalent in NI (7.77 ~ 12.72%) than PI (4.12 ~ 11.19%). *Blautia* and *Mediterraneibacter gnavus* group of *Lachnospiraceae* exhibited higher abundances in PI (0.14 ~ 1.17% and 1.04 ~ 3.41%) than NI (0.14 ~ 0.94% and 0.83 ~ 3.32%).

### 3.5 Changes in SCFAs and ethanol

**Figure 6:**
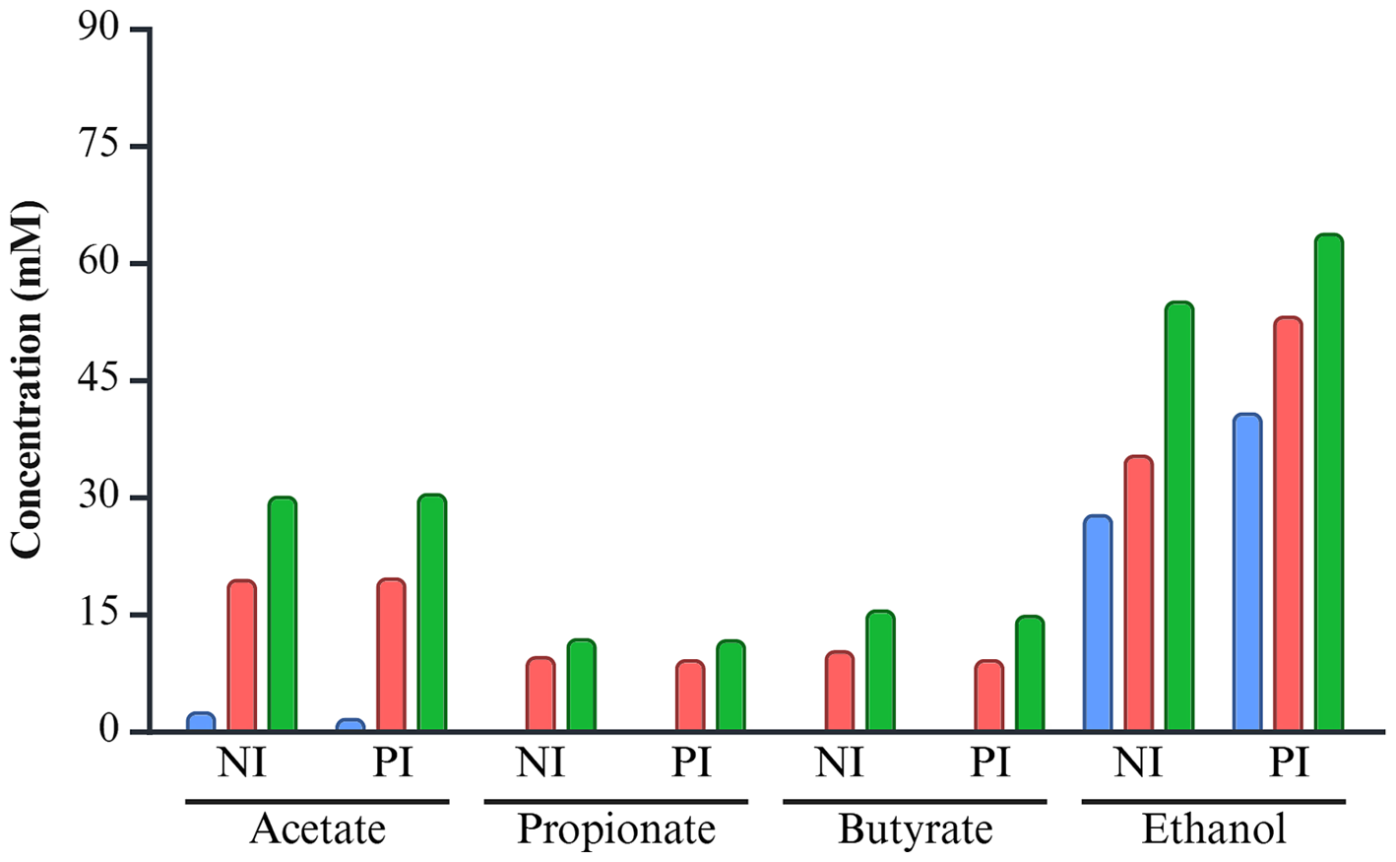
The concentration of short-chain fatty acids and ethanol of *L. monocytogenes* infected and non-infected pooled fecal samples during fermentation. The color of each bar chart represents the different incubation times: 6 h (blue), 12 h (red), and 24 h (green). NI, non-infected; PI, post-infected.

Acetate and propionate concentrations were comparable between the NI and PI groups, with acetate ranging from 0 to 30.14 mM in NI and 0 to 30.47 mM in PI, and propionate ranging from 0 to 11.74 mM in NI and 0 to 11.88 mM in PI (Figure 6). Butyrate levels were slightly higher in NI (0 ~ 15.55 mM) compared to PI (0 ~ 14.87 mM). In contrast, ethanol production was elevated in the PI group, ranging from 39.07 to 63.82 mM, compared to 27.78 to 55.13 mM in the NI group. Over time, the concentration of both SCFAs and ethanol increased progressively throughout the incubation period.

## 4 Discussion

*Listeria monocytogenes* is a significant public health and food safety concern because of its virulence and resistance against various environmental stresses (Muchaamba et al., 2022). Its infectious dose can be influenced by the human innate immune system, which plays a crucial role in determining the severity of infection (Guo et al., 2023; Koopmans et al., 2023). During the gastrointestinal passage, *L. monocytogenes* must endure the stressful environment, with its survival capability varying by strain, isolate origin, and serotype (Ramalheira et al., 2010; Zou et al., 2024). Survived *L. monocytogenes* that bypass host’s defense systems must encounter gut microbiota, which competes for energy sources and secretes antibacterial compounds (Kamada et al., 2013). In immunocompromised individuals, *L. monocytogenes* can establish prolonged colonization in the cecum and colon, thereby altering the gut microbiota profile (Koopmans et al., 2023). Although several studies have explored the changes in gut microbiota due to *L. monocytogenes* infection, specific interactions between bacteria remain poorly understood (Becattini et al., 2017; Guo et al., 2023; Wolter et al., 2021). In this study, we aimed to investigate the survival of *L. monocytogenes* during digestion, assess its pathogenic risk, and determine its impact on gut microbiota and metabolite profiles.

Among the 14 serotypes of *L. monocytogenes*, 92 ~ 95% of the clinical isolates belong to serotypes 4b, 1/2a, and 1/2b (Koopmans et al., 2023). Serotypes 4b and 1/2a exhibit higher resistance to environmental stresses, including acidity, than serotypes 4a and 1/2c (Chakravarty et al., 2021; Jiang et al., 2010; Zou et al., 2024). Additionally, clinical isolates exhibited greater resistance to acidic and bile stresses compared to food isolates (Ramalheira et al., 2010). *L. monocytogenes* F2365 (serotype 4b) and NCCP 14714 (serotype 1/2b) were clinical isolates, while ATCC 7644 (serotype 1/2c) was isolated from food (Koopmans et al., 2023). *L. monocytogenes* F2365 and NCCP 14714 showed significantly higher resistance in the simulated gastric phase (pH 2.0) than ATCC 7644. Acidic stress adaption is regulated by the stress response factor σ^B^, encoded by the *rsbRSTUVWX* and *sigB* genes (Gahan and Hill, 2014; Guerreiro et al., 2022). The *rsbS* gene plays a critical role in stress signaling, as it mediates the phosphorylation and activation of the stressosome (Guerreiro et al., 2022). Notably, a *ΔrsbS* mutant strain exhibited reduced acid tolerance, falling below detection limits after just 15 min of exposure to pH 5.0 (Guerreiro et al., 2022). *L. monocytogenes* ATCC 7644 has an adenine deletion in the *rsbS* gene with a premature stop codon, resulting in reduced survival under acidic conditions. This characteristic suggests the need for further research to explore the mechanisms underlying this phenomenon. Previous studies have documented the presence of multiple *L. monocytogenes* strains in a single food sample, as well as multi-strain involvement in listeriosis outbreaks (Zilelidou and Skandamis, 2018). In addition to the individual strains, a five-strain cocktail was evaluated to reflect potential real-world contamination scenarios involving multiple serotypes. The cocktail exhibited greater resistance than the more acid-sensitive individual strains, such as ATCC 7644, but showed lower resistance than F2365 and NCCP 14714. Although inter-strain interactions among *L. monocytogenes*, such as quorum sensing, bacteriocin production, or biofilm cooperation, may enhance virulence or survival, the overall survival observed in the cocktail can be attributed primarily to the intrinsic resistance of the more tolerant strains (Zilelidou and Skandamis, 2018).

The human colon contains nearly 11 to 12 log CFU/g of bacteria, comprising 500 to 1,000 species (Rizzatti et al., 2017). Factors such as diet, illness, and antibiotic treatment can significantly impact microbial diversity and often result in gut dysbiosis (Rogers et al., 2021). In the present work, alpha and beta diversity of the gut microbiota showed three distinct phases regardless of infection status. A significant decline in the diversity was observed within the first 6 h, followed by a gradual recovery, highlighting a rebalance within the microbial ecosystem and the resilience of the gut microbiota after exposure to stress. A decline can be attributed to dysbiosis induced by the initial fecal processing step, resulting in unfavorable conditions (Rogers et al., 2021). Pseudomonadota decreased over time, which includes various human pathogens, such as *Shigella*, *Escherichia*, *Salmonella*, *Yersinia*, and *Helicobacter* (Rizzatti et al., 2017). Conversely, Fusobacteriota showed a continuous increase, suggesting that the gut microbiota had reached an alternate stable state which was different from its baseline status.

Gut dysbiosis can increase susceptibility to *L. monocytogenes* infection. Once established, listerial infections may exacerbate the condition by further disrupting the microbial community structure (Bäumler and Sperandio, 2016; Becattini et al., 2017). In C57BL/6 mice, *L. monocytogenes* significantly decreased Chao1 and Shannon indices, indicating a reduction in microbial diversity (Alam et al., 2021; Keane et al., 2023). Similarly, a decrease in evenness was found in the macaque monkey, although the difference was insignificant (Hugon et al., 2023). In this study, *L. monocytogenes* infection resulted in decreased richness and increased evenness, consistent with a reduction in specific taxa and the reorganization of community structure. Furthermore, PCA analysis of weighted distances also revealed distinct clustering between non-infected and infected groups, highlighting differences in the microbial community composition. The observed differences in gut microbiota diversity can be, in part, attributed to bacteriocins produced by *L. monocytogenes*, such as listeriolysin S (LLS) and Lmo2776 (Lee, 2020). *L. monocytogenes* ATCC 7644 and F2365 have the Lmo2776 synthesis operon and Lmo2776 expressed under *in vitro* conditions (Rolhion et al., 2019). Lmo2776 specifically targeted *Prevotella* spp. and *Segatella copri* (formerly *Prevotella copri*) (Rolhion et al., 2019). *L. monocytogenes* NCCP 14714 and F2365 contain the LLS synthesis cluster. LLS is expressed in gastrointestinal tracts *in vivo* and decreases the abundance of *Allobaculum* and *Alloprevotella* in mice (Quereda et al., 2016; Quereda et al., 2017). The antimicrobial activities of these *L. monocytogenes*-derived bacteriocins may suppress the richness of specific microbial taxa. Conversely, the absence of such bacteriocins could facilitate the proliferation of other microbial species by relieving competitive pressures within the gut environment.

In this study, *L. monocytogenes* infection led to a decrease in Bacillota, while Bacteroidota was increased, consistent with the findings from the BALB/c mice study by Guo et al. (2023), though opposite results were reported in C57BL/6 mice model (Alam et al., 2021). Bacteroidota has been linked to inflammation, suggesting that *L. monocytogenes* can modulate inflammation by altering the gut microbial composition (Guo et al., 2023; Rogers et al., 2021). The reduction and delayed recovery of Bacillota may weaken gut barrier function and impair immune regulation, heightening susceptibility to pathogens and promoting inflammation (Rogers et al., 2021). *L. monocytogenes* infection led to an increase in Pseudomonadota in the BALB/c mice, which contradicts the findings of this study (Guo et al., 2023). Elevated levels of Pseudomonadota was reported in pregnant individuals, who are at the highest risk for listeriosis (Becattini et al., 2017; Koopmans et al., 2023). However, little research has been conducted on the specific relationship between Pseudomonadota and *L. monocytogenes*.

Several bacterial groups known for their protective roles against intestinal pathogens showed increased abundance following *L. monocytogenes* infection in this study. *Bacteroides* spp. contribute to intestinal homeostasis by degrading mucin and supporting nutrient availability for other microbes (Zafar and Saier Jr, 2021). They also interact with the host immune system and compete with pathogens (Wexler, 2007; Zafar and Saier Jr, 2021). In murine models, *L. monocytogenes* infection altered *Bacteroides* populations: *B. caccae* increased while *B. ovatus* decreased in BALB/c mice, and in C57BL/6 mice, *B. uniformis* and *Bacteroidaceae* decreased while *B. acidifaciens* increased (Alam et al., 2021; Guo et al., 2023; Las Heras et al., 2019; Wolter et al., 2021). The observed increase in *Bacteroides* may have contributed to the reduction of Pseudomonadota in our study, potentially through colonization resistance mechanisms. In particular, *B. thetaiotaomicron* has been shown to directly inhibit the colonization of *E. coli* and *Salmonella*, both members of Pseudomonadota, supporting the proposed role of *Bacteroides* in colonization resistance (Buffie and Pamer, 2013). *Bifidobacterium* spp. also play a well-established role in inhibiting pathogen colonization. They improve host outcomes in infections by *E. coli* O157: H7 and *Clostridium perfringens*, and can inhibit *L. monocytogenes* EGDe invasion by 60–90% through secreted proteinaceous factors (Corr et al., 2007; O'Callaghan and Van Sinderen, 2016). The increased abundance of *Bifidobacterium* following listerial infection may be related to *L. monocytogenes* mitigating oxidative stress on *B. bifidum* through neutralization of reactive oxygen species (Yu et al., 2020). *Lachnospiraceae*, also known as *Clostridium* cluster XIVa, are fiber-degrading bacteria that produce SCFAs and help maintain gut barrier function (Vacca et al., 2020). Within this family, *Blautia producta* has demonstrated anti-listerial activity by inhibiting *L. monocytogenes* propagation (Becattini et al., 2017). Increased abundance of *Lachnospiraceae* has been observed in infected C57BL/6 mice fed a high-fat diet and in aged C57BL/6 mice (Alam et al., 2021; Las Heras et al., 2019), and a positive correlation (*ρ* = 0.33) was reported between *Lachnospiraceae* and *L. monocytogenes* abundance in human listeriosis patients (Hafner et al., 2021). These findings align with our observation of elevated *Lachnospiraceae* following infection. The increased abundance of *Bacteroides*, *Bifidobacterium*, and *Lachnospiraceae*, which are known to protect against *L. monocytogenes*, following infection may reflect an intrinsic compensatory mechanism of the human gut microbiota to restore microbial balance in response to pathogenic challenge (Becattini et al., 2017; Buffie and Pamer, 2013; Corr et al., 2007). This observation supports the hypothesis that individuals with a diverse and resilient gut microbiota are better protected against listeriosis (Guo et al., 2023; Hafner et al., 2021; Wolter et al., 2021).

The direct impact of SCFA on *L. monocytogenes* has been unknown, however, SCFAs can regulate the host’s immune system and enhance gut barrier function by strengthening tight junctions (Becattini et al., 2017; Rogers et al., 2021). Acetate and propionate exhibited a synergistic effect with nisin, produced by *Lactococcus lactis*, potentially contributing to shifts in the bacterial profile (Rodpan et al., 2022). SCFAs produced by beneficial microbes such as *Bifidobacterium* could lower intestinal pH, thereby creating an unfavorable environment for opportunistic pathogens (O'Callaghan and Van Sinderen, 2016). The pH in this study decreased to 6.98 due to the buffering capacity of MiPro medium, limiting the ability to assess microbial changes directly related to pH fluctuations.

Acetate, propionate, and butyrate are detected in the human colon and stool at a 3:1:1 ratio, with total SCFA concentrations ranging from 20 to 70 mM in the distal colon (Den Besten et al., 2013). Comparable levels were observed in this study, with ~30.14 mM acetate, ~11.88 mM propionate, and ~15.55 mM butyrate. Although aformentioned bacteria such as *Bacteroides*, *Bifidobacterium*, and *Lachnospiraceae* are known contributors to SCFA production, no significant differences in SCFA levels were observed between infected and non-infected groups (O'Callaghan and Van Sinderen, 2016; Vacca et al., 2020). *Listeria* infection increased ethanol levels, a primary metabolite associated with bacteria such as *B. thetaiotaomicron*, *Bifidobacterium*, and *M. gnavus* group (Crost et al., 2018; Elshaghabee et al., 2016; Yamaguchi et al., 2018). Although many gut microbiota are capable of producing ethanol, its elevated levels during the infected state are linked to an increased abundance of these bacteria. Ethanol can disrupt epithelial cells and weaken tight junctions. This “leaky gut” condition allows harmful substances, such as endotoxins, to penetrate the bloodstream, triggering systemic inflammation and potentially exacerbating *Listeria* infection (Chen et al., 2022). Since most gut microbiota cannot metabolize ethanol, it is predominantly converted to acetate through host metabolic pathways (Martino et al., 2022). Ethanol consumption increases Bacteroidetes more through elevated acetate levels, an ethanol metabolite, than through ethanol itself (Martino et al., 2022). As this *in vitro* model excludes host interactions, predicting acetate accumulation from ethanol metabolism and its impact on the gut microbiota remains challenging. Therefore, the observed increase in Bacteroidetes cannot be solely attributed to ethanol.

This study highlights the intricate interactions between *L. monocytogenes* and the gut microbiota, emphasizing the pathogen’s ability to survive and adapt within the gastrointestinal environment. Our study demonstrates that clinical *L. monocytogenes* strains exhibit greater resistance to the human digestive process than food-derived strains. *L. monocytogenes* was observed to cause minimal to no significant changes in the gut microbiota diversity, consistent with previous studies (Becattini et al., 2017; Hugon et al., 2023; Keane et al., 2023; Las Heras et al., 2019; Li et al., 2019). Hugon et al. (2023) reported that *L. monocytogenes* alone did not induce dysbiosis; however, listeriosis may contribute to gut microbiota alterations under specific conditions, such as pregnancy. Comparative studies using macaque monkeys, BALB/c mice, and C57BL/6 mice have reported varying results, highlighting discrepancies between animal models and human microbiome data (Alam et al., 2021; Guo et al., 2023; Hafner et al., 2021; Hugon et al., 2023). These differences may stem from defense mechanisms against pathogens and variations in the host’s immune system. The administration of *Akkermansia muciniphila* to mice has been shown to decrease susceptibility to *L. monocytogenes* (Keane et al., 2023). This protective effect was not directly attributed to changes in gut microbiota composition or the bacterium itself but rather through interactions with the host’s immune system, indicating that the inhibition of *L. monocytogenes* by gut microbiota may be more intricate than previously understood (Keane et al., 2023). LLS expressed by *L. monocytogenes* has been found to have no effect on human eukaryotic cells but exhibits antimicrobial activity against prokaryotes, suggesting that *Listeria* can directly influence gut microbiota composition (Quereda et al., 2017). This study primarily focused on the interplay between human gut microbiota and *Listeria* and did not account for the potential involvement of the immune system. The findings provide valuable insights into understanding complex mechanisms underlying gut microbiota-pathogen interactions. Notably, *Listeria* infection was associated with an increased abundance of bacteria such as *Bacteroides*, *Bifidobacterium*, and the *M. gnavus* group, which are known ethanol producers. Elevated ethanol levels may compromise epithelial barrier integrity to a leaky gut that facilitates the translocation of harmful substances, exacerbates inflammatory responses, and further complicates conditions like listeriosis. Although ethanol has not been previously recognized as a metabolic biomarker in *Listeria* infection studies, the unexpected increase observed in this study highlights the need for further investigation into its role in gut function and host health. Due to the use of pooled samples, correlation analyses between specific taxa and ethanol production were not feasible, limiting direct functional inference. Future studies with larger sample sizes and individual-level measurements will be required to validate these observations.

## Data Availability

The datasets presented in this study can be found in online repositories. The names of the repository/repositories and accession number(s) can be found: https://www.ncbi.nlm.nih.gov/, PRJNA1254141.
