# Supplementary material for: Exploring the fate of Listeria monocytogenes in an in vitro digestion and fecal fermentation model: insights into survival during digestion and interaction with gut microbiota
Source: Front Microbiol. 2025 Jul 23;16:1616720. doi: 10.3389/fmicb.2025.1616720 (PMC12325261; doi:10.3389/fmicb.2025.1616720)
Supplement: Supplementary file 1 [file Data_Sheet_1.docx]

Supplementary Material

# Supplementary Figures and Tables

## Supplementary Figures


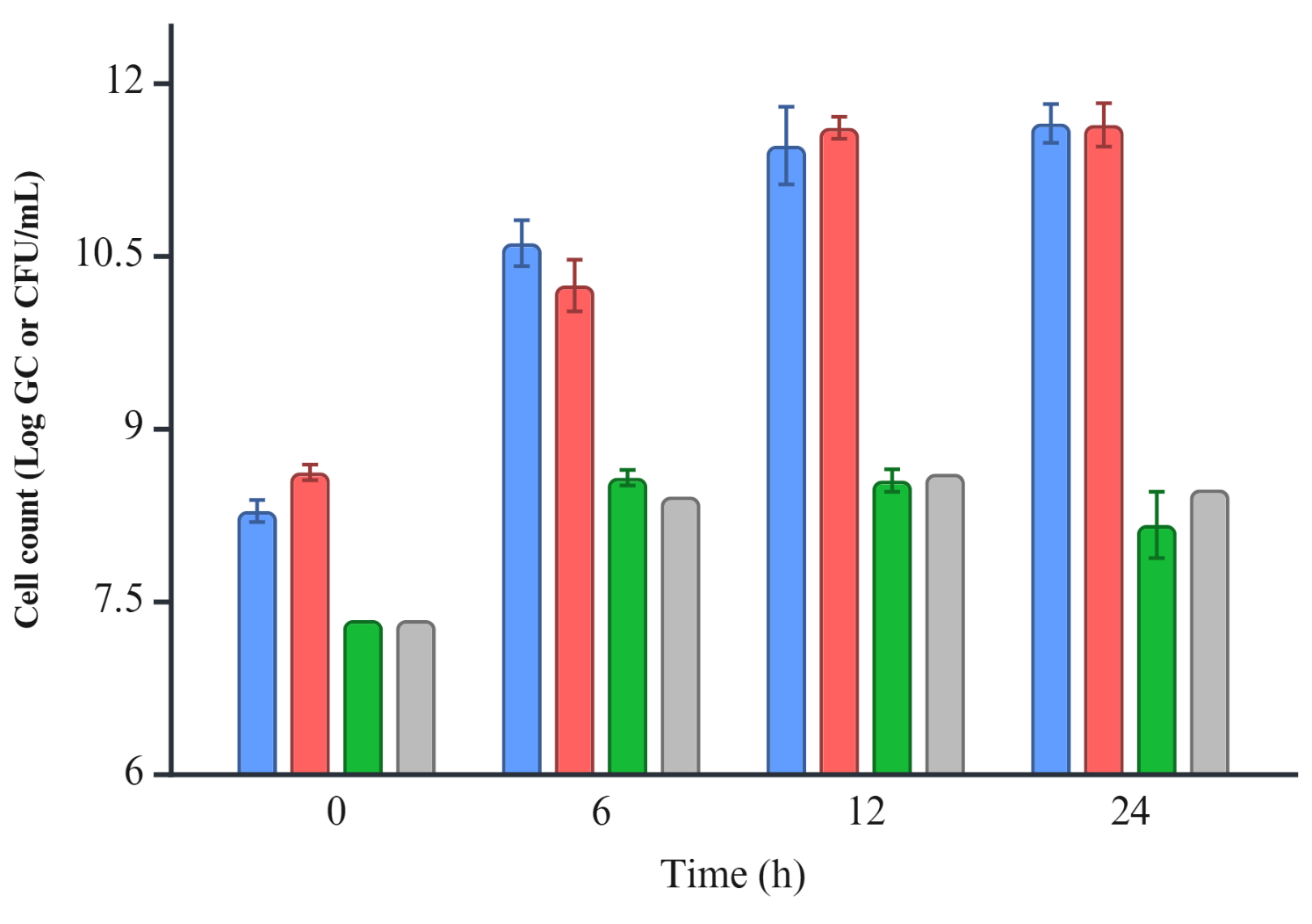


**Supplementary Figure S1.** Bacterial concentrations during fecal fermentation: non-infected gut microbiota (blue), post-infected gut microbiota (red), *L. monocytogenes* in the post-infected condition (green), and the concentration of *L. monocytogenes* in MiPro medium (gray) during incubation. No significant differences were observed between non-infected and post-infected gut microbiota, as well as *L. monocytogenes* at any time point (*p* > 0.05).

**
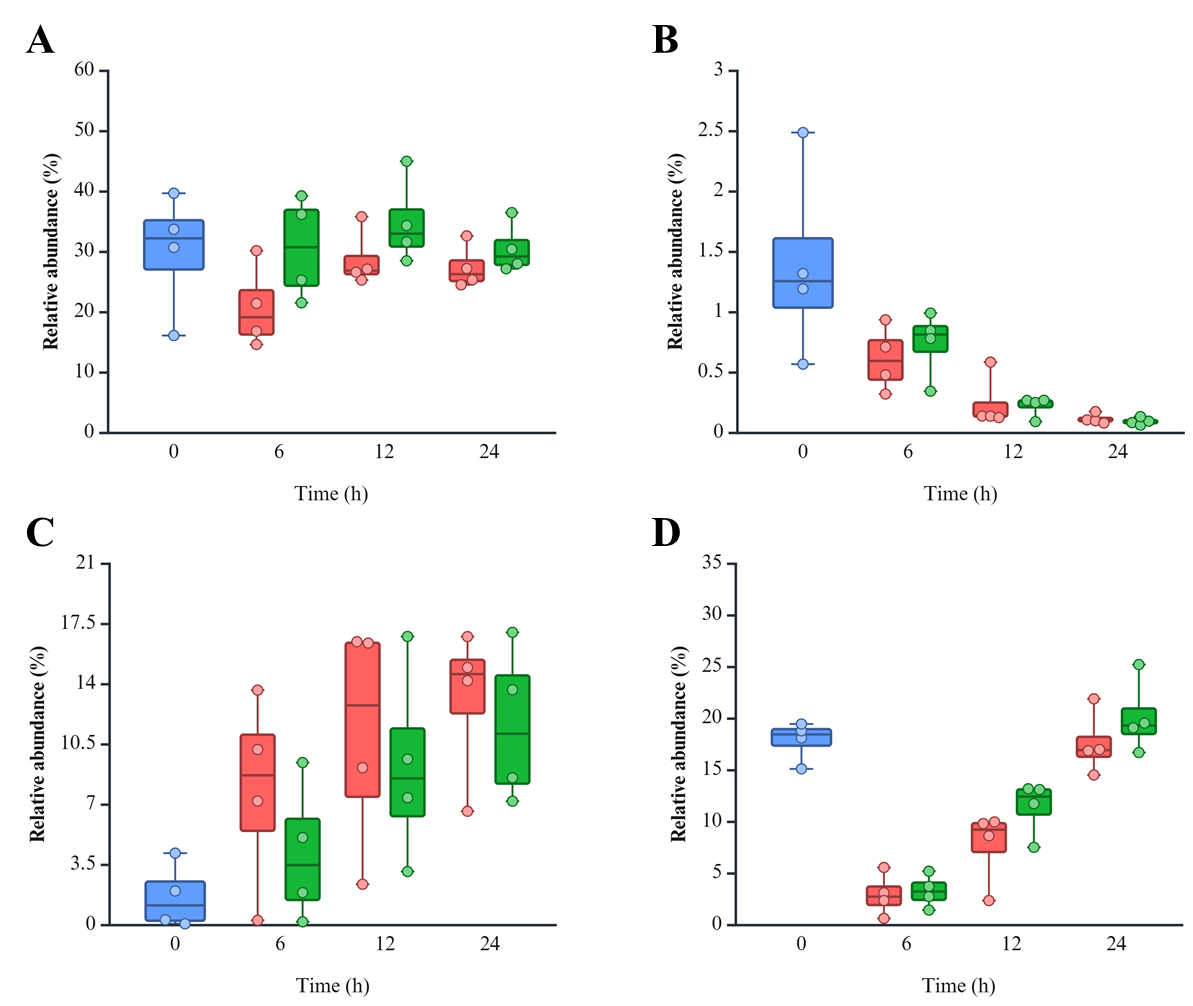
**

**Supplementary Figure S2.** Relative abundance of four families of interest: **(A)** *Bacteroidaceae*, **(B)** *Bifidobacteriaceae*, **(C)** *Erysipelotrichaceae*, and **(D)** *Lachnospiraceae*. The color of each bar chart represents the different infection conditions: control (blue), non-infected (red), and post-infected (green). Each dot represents the value for each sample.
